# Improved Biomedical Properties of Polydopamine-Coated Carbon Nanotubes

**DOI:** 10.3390/mi12111280

**Published:** 2021-10-20

**Authors:** Sahin Demirci, Mehtap Sahiner, Selin Sagbas Suner, Nurettin Sahiner

**Affiliations:** 1 Department of Chemistry, Faculty of Sciences and Arts, Canakkale Onsekiz Mart University, Terzioglu Campus, Canakkale 17100, Turkey; sahindemirci@gmail.com (S.D.); sagbasselin@gmail.com (S.S.S.); 2 Nanoscience and Technology Research and Application Center, Canakkale Onsekiz Mart University, Terzioglu Campus, Canakkale 17100, Turkey; 3 Faculty of Canakkale School of Applied Science, Canakkale Onsekiz Mart University, Terzioglu Campus, Canakkale 17100, Turkey; sahinerm78@gmail.com; 4 Department of Chemical and Biomolecular Engineering, University of South Florida, Tampa, FL 33620, USA; 5 Department of Ophthalmology, Morsani College of Medicine, University of South Florida, 12901 Bruce B Downs B. Downs Blv., MDC 21, Tampa, FL 33612, USA

**Keywords:** carbon nanotubes, polydopamine, biomedical, coating, PDA@CNT

## Abstract

Carbon nanotubes (CNTs) due to their outstanding mechanical, thermal, chemical, and optical properties were utilized as a base material and were coated with polydopamine (PDA) (PDA@CNT) via the simple self-polymerization of dopamine (DA). Then, PDA@CNT coatings of up to five layers were examined for potential biomedical applications. The success of multiple coating of CNTs with PDA was confirmed via increased weight loss values with the increased number of PDA coatings of CNTs at 500 °C by thermogravimetric analysis. The surface area of bare CNTs was measured as 263.9 m^2^/g and decreased to 197.0 m^2^/g after a 5th coating with PDA. Furthermore, the antioxidant activities of CNT and PDA@CNTs were determined via total flavonoid content (TFC), total phenol content (TPC), and Fe(III)-reducing antioxidant power (FRAP) tests, revealing the increased antioxidant ability of PDA@CNTs with the increasing numbers of PDA coatings. Moreover, a higher inhibition percentage of the activity of the alpha-glucosidase enzyme with 95.1 ± 2.9% inhibition at 6 mg/mL PDA-1st@CNTs concentration was found. The CNT and PDA@CNTs exhibited blood compatibility, less than a 2.5% hemolysis ratio, and more than 85% blood clotting indexes. The minimum inhibition concentration (MIC) of PDA-5th@CNTs against *E. coli* and *S. aureus* bacteria was determined as 10 mg/mL.

## 1. Introduction

Carbon nanotubes (CNTs) are 1D nanostructures derived from the rolling of one or more than one graphene sheets, which are called single-walled and multi-walled CNTs, respectively [1,2,3]. It was reported in the literature that the size of CNTs varies up to hundreds of micrometers in length and few tens of nm in diameters [4,5,6]. CNTs have stimulated the curiosity of researchers across many disciplines to be utilized within many fields in semiconductors, sensors, energy conversion, storage systems, and water purification [5,7,8,9] because of their unusual physical and chemical characteristics e.g., high thermal and mechanical stability, large surface area, high aspect ratio, electrical and chemical inertness [10,11,12]. On the other hand, the Van der Waals interactions of the bare CNTs causing the drawback of dispersibility within the polymer matrix can impede the processability and the ability of widespread use of pure CNTs in versatile composite applications [13,14].

Nature’s dimensionality in nanoscience has understandably raised interest for the use of nanomaterials in the biomedical industry with the possibility of viable applications in medicine [15,16]. As a result of its established unique characteristics, carbon-based nanotechnology is swiftly emerging as a platform for a wide range of biological applications [17,18,19,20,21,22] following Iijima’s discovery of CNTs [2]. However, because of their extremely hydrophobic nature, the inability of CNTs to disperse in aqueous environments remains the most significant challenge in the biological and medicinal application of CNTs [16].

Coating technology is commonly used in the functionalization of materials [23,24,25] for different purposes, and various types of coating process can be applied depending on the types of materials [26,27,28,29]. By appropriately altering the surface properties of CNTs, the major problems of agglomerating propensity, poor dispersion, and weak interfacial bonding properties of CNTs may be overcome [30]. There are many studies that examine the functionalization of CNTs to circumvent the dispersibility problem of carbon-based materials, especially of CNTs [31,32,33,34,35,36,37]. Depending on the sort of interactions that take place between active molecules and carbon atoms on CNTs, the functionalization procedures may be separated into chemical functionalization (or covalent functionalization) and physical modification (or non-covalent functionalization) [30]. The π–π conjugation system on the surface of the CNTs is destroyed after chemical treatment due to the change in hybridization from sp^2^ to sp^3^ [31,33,35], whereas researchers also reported some functionalization approaches that keep the electronic properties of CNTs even after increasing the amount of sp^3^ carbons upon functionalization can be retained [38,39,40]. As a result, non-covalent functionalization is a hot topic in these fields. The π–π conjugation system in the surface graphene layer of CNTs is not disturbed in this manner, and their original characteristics are expected to be preserved. The Van der Waals force of contact between the tube wall and the interacting molecules causes a non-covalent functionalization of CNTs [30,32,33].

The self-polymerization of dopamine, a bio-inspired mussel adhesive protein, results in polydopamine (PDA) coatings, which are by far one of the finest advancements in the surface modification of materials [7]. In recent years, the coatings of CNTs with PDA have been attracted by many researchers. For example, PDA coating of CNTs has been reported to improve their metal ion removal ability [11], polymer electrolyte membranes for direct methanol fuel cells [13], high permittivity and low dielectric loss [41], controlling cell behavior [42], protein separation [7], etc. It was also reported that the coating of CNTs with PDA can significantly improve their cytocompatibility [43,44,45].

Here, multiple coatings of CNTs with PDA as PDA@CNT was carried out via the processing of self-polymerization of DA in a 10 mM TRIS buffer at pH 8.5 up to five layers. The PDA@CNTs were characterized by FT-IR, UV-Vis, TGA, SEM, BET, and XRD measurements. The antioxidant ability of PDA@CNTs and the effect of coating numbers of PDA on CNTs on the antioxidant ability were investigated via three different assays such as total flavonoid content (TFC), total phenol content (TPC), and Fe(II)-reduced antioxidant power (FRAP). On the other hand, the effect of multiple PDA coatings on CNTs on the blood compatibility, enzyme inhibition, and antibacterial properties of prepared PDA@CNTs in comparison to bare CNT was investigated. The antibacterial activity of PDA@CNT on Gram-negative *E. coli* and Gram-positive *S. aureus* bacteria was also investigated via the macro-dilution method.

## 2. Materials and Methods

### 2.1. Materials

Multi-walled carbon nanotubes (CNT, 98%, O.D × I.D × L 10 ± 1 nm × 4.5 ± 0.5 nm × 3–6 µm TEM, Sigma Aldrich, Milwaukee, WI, USA) and dopamine hydrochloride (DA, 98%, Sigma, Milwaukee, WI, USA) were used as received. TRIS (99%, Sigma Aldrich, Milwaukee, WI, USA) was used in the preparation of buffer solution to the polymerization of DA during the coating process of CNTs. Sodium nitrite (NaNO_2_, extra pure, Merck, Darsmstadt, Germany) and aluminum chloride hexahydrate (AlCl_3_.6H_2_O, 99%, Alfa Aesar, Ward Hill, MA, USA) were used for the total flavonoid test. Folin–Ciocalteau’s phenol reagent (FC) (Sigma-Aldrich, Milwaukee, WI, USA) was used for the TPC test. Gallic acid (GA) (97.5–102.5%, Aldrich, Milwaukee, WI, USA) was used as a reference antioxidant polyphenol. 2,4,6-Tri(2-pyridyl)-s-triazine (TPTZ, Sigma Aldrich), sodium acetate anhydrous (99%, Fisher, Atlanta, GA, USA), hydrochloric acid (37%, Sigma, Atlanta, GA, USA), iron(III) chloride hexahydrate (FeCl_3_∙6H_2_O, ≥99%, Acros, Geel, Belgium), and iron(II) chloride tetrahydrate (FeCl_2_∙4H_2_O, ≥99%, Sigma Aldrich, Milwaukee, WI, USA) were used for ferric (III)-reducing antioxidant power (FRAP) assay test. α-Glucosidase from Saccharomyces cerevisiae (100 unit/mg, Sigma Aldrich, Milwaukee, WI, USA), 4-nitrophenyl-α-D-glucopyranose (4-NPG, 99%, Acros, Geel, Belgium), and potassium phosphate monobasic (98–100.5%, Sigma Aldrich, Milwaukee, WI, USA) were used for enzyme studies.

Bacteria growth media were used as received, including nutrient agar (NA, Fisher Scientific) and nutrient broth (NB, Fisher Scientific, Atlanta, GA, USA). Microbiologics provided two bacterial strains: *E. coli*-ATCC 8739 (KWIK-STIK TM) and *S. aureus* ATCC 6538 (KWIK-STIK TM). Acros Organics (Geel, Belgium) provided gentamicin sulfate (>590 IU/mg gentamycin) as an antibiotic.

### 2.2. Coating of CNTs with PDA

The coating of CNTs with PDA was carried out by self-polymerization of DA in TRIS buffer [46]. First, the CNTs were washed for 24 h with an ethanol/water (50:50, *v*/*v*) mixture to clean the CNTs from unexpected residues that may have come from the CNTs preparation process. The cleaning of CNTs was carried out via the stirring of 10 g CNT in 1 L of ethanol/water (50:50, *v*/*v*) mixture at room temperature and at a 1000 rpm mixing rate for 24 h. After that, the washed CNTs were precipitated via the centrifugation of an ethanol/water mixture at 10,000 rpm and dried with a freeze-dryer at −86 °C and 0.011 mbar. After that, 10 g of washed and dried CNTs were dispersed into freshly prepared 500 mL of DA solution at 2 mg/mL concentration in 10 mM TRIS buffer at pH 8.5. Then, the self-polymerization reaction of DA was performed for 6 h stirring at 1000 rpm to coat the CNTs with PDA. Finally, the PDA coating on CNTs (PDA-1st@CNT) was separated via 10 min centrifugation at 10,000 rpm and washed with water twice. A certain amount of PDA-1st@CNT (2 g) was separated, and the remaining 8 g of PDA-1st@CNT was coated with PDA via the same coating procedure. This coating process was realized 5 times consecutively, and 2 g of PDA@CNTs at each coating number (PDA-2nd@CNT, PDA-3rd@CNT, PDA-4th@CNT, and PDA-5th@CNT) were separated, washed, dried, and stored in closed tubes for characterization and further usages. After each coating, for example, the collected PDA-1st@CNTs were washed with water twice and dried in a freeze-dryer. Then, the same process was applied to PDA-2st@CNTs to the 3rd coating with PDA and so on. The drying process of samples was carried out via a freeze-dryer.

### 2.3. Characterizations of PDA@CNTs

The functional groups of PDA@CNTs were identified using Fourier Transform Infrared Irradiation (FT-IR, Spectrum, Perkin Elmer Instruments, Akron, OH, USA) spectroscopy. The FT-IR spectra of all powder products were obtained via the potassium bromide (KBr) pellet method and recording the spectra in the range of 4000–400 cm^−1^ with 16 repetitive scan runs.

The percentage of coated PDA on CNTs was calculated by thermogravimetric analysis using a Thermogravimetric Analyzer (TGA, SII TG/DTA6300, Exstar, Seiko Ins. Corp, Chiba, Japan), which involved heating a 3–5 mg PDA@CNTs specimen from 100 to 500 °C at a rate of 10 °C/min in a nitrogen environment.

Scanning electron microscopy (SEM, SU-70, Hitachi, Japan) images of bare CNT and PDA-5th@CNTs were taken after the attachment of samples on SEM stubs. The samples were covered with Pd in a few nanometers under vacuum, and images were taken under 20.0 kV operating voltage.

X-Ray diffraction powder patterns of CNT and PDA@CNTs were recorded on a Bruker D8 Advance diffractometer in the scanning range of 5–80° (2*θ*) using a Cu Kα radiation at 40 kV and 40 mA at 1.5418 Å wavelength.

The change of surface area and the porosity properties of CNT and PDA@CNTs were investigated via employing a surface area and porosity analyzer (TriStar II, Micromeritics, Norcross, GA, USA) with N_2_ adsorption/desorption measurements and BET and BJH methods. Before analysis, CNT-based samples were degassed with N_2_ gas for 12 h before analysis, and N_2_ gas adsorption/desorption curves were obtained under a liquid nitrogen environment.

### 2.4. Antioxidant Assays

Antioxidant capacities of bare CNT, PDA-1st@CNT, PDA-3rd@CNT, and PDA-5th@CNT were determined via three different antioxidant assays such as the total phenol content (TPC), total flavonoid content (TFC), and ferric-reducing antioxidant power (FRAP) in accord with the literature [47]. The sample solutions in water at 2 mg/mL concentrations were prepared for TFC and TPC tests, and pH 3.6 acetate buffer was used for the FRAP test. Before starting all the tests, the samples were suspended in solutions and were sonicated at room temperature for 10 min.

#### 2.4.1. Total Flavonoid Content Assay

The total flavonoid content (TFC) of CNTs and PDA@CNTs was calculated by using the technique reported by our groups [47] with minor changes. Briefly, 0.5 mL of sample solutions at 2 mg/mL concentrations, 2 mL of DI water, and 0.15 mL of 5% NaNO_2_ aqueous solution were mixed in a 10 mL tube. After 5 min, 0.15 mL of 10% AlCl_3_ aqueous solution was added, which was followed by 1 mL of 1 M NaOH aqueous solution after another 5 min. After 15 min, the absorbance of this solution was measured using UV-Vis spectroscopy at 405 nm against a previously established calibration of TFC values of the gallic acid standard. The total flavonoid content was reported as µg/mL gallic acid equivalence.

#### 2.4.2. Total Phenol Content Assay

The Folin–Ciocalteu (FC) reaction was used to determine the total phenol content (TPC) of CNTs and PDA@CNTs, as described earlier [47]. For 4 min, 0.1 mL of this sample solution at a concentration of 2 mg/mL was reacted with 1.25 mL of 0.2 N FC reagent aqueous solution. Next, 1 mL of 0.7 M Na_2_CO_3_ aqueous solution was added to the reaction, and it was kept at room temperature for 2 h in the dark. Then, using UV-Vis spectroscopy, the absorbance values of the solutions were measured at 760 nm against a previously produced calibration of TPC values of gallic acid standard, and the findings were provided as µg/mL gallic acid equivalency of total phenol content.

#### 2.4.3. Ferric-Reducing Antioxidant Power (FRAP) Assay

The ferric-reducing antioxidant power (FRAP) test was used to assess the antioxidant potential of CNTs and PDA@CNTs via the reduction process of modified ferric to ferrous [47]. In the employed method, 2.5 mL of 20 mM FeCl_3_ solution in 0.3 M acetate buffer and 10 mM tripyridyl triazine (TPTZ) solution in 2.5 mL of 40 mM HCl aqueous solution were added in 25 mL of pH 3.6 acetate buffer (0.3 M) to prepare the Fe(III)-TPTZ complex. UV-Vis spectroscopy was used to determine the absorbance of this complex solution at 595 nm. Then, in a 0.3 M acetate buffer, 20 mL of CNTs and PDA@CNTs at a concentration of 2 mg/mL were treated with 3 mL of Fe(III)-TPTZ complex solution. Using UV-Vis spectroscopy, the absorbance of this complex solution was measured at 595 nm after 4 min. The discrepancies in the absorbance values of Fe(III)-TPTZ complex solution without and with the antioxidant sample against the calibration of the Fe(II)-TPTZ complex were used to compute the ferric-reducing capacity of CNTs and PDA@CNTs, and FRAP values were represented in µmol Fe(II) reduced.

### 2.5. Blood Compatibility Assay

The hemolysis and blood-clotting analyses of CNT, PDA-1st@CNT, PDA-3rd@CNT, and PDA-5th@CNT were studied to determine the blood compatibility of materials using the technique given by Zamani et al. with minor modifications [48]. Fresh blood was drawn from a healthy volunteer and promptly put into EDTA-containing tubes in accord with clearance from the Canakkale Onsekiz Mart University’s Human Research Ethics Committee (011-KAEK-27/2020-E.2000045671).

#### 2.5.1. Hemolysis Assay

Fresh blood, 4 mL was diluted with 5 mL of 0.9% NaCl aqueous solution for the hemolysis procedure. Separately, 5 mg of CNT, PDA-1st@CNT, PDA-3rd@CNT, and PDA-5th@CNT were put in tubes containing 10 mL of 0.9% NaCl solution (final concentration is 0.5 mg/mL). Then, 200 μL of diluted blood was gently injected to these tubes to enable the blood to make contact with the CNT, PDA-1st@CNT, PDA-3rd@CNT, and PDA-5th@CNTs. After 1 h of incubation at 37 °C, the CNTs/PDA@CNTs–blood solution was centrifuged at 100 g for 5 min to separate undamaged erythrocytes. To determine the hemolysis ratio of materials, the absorbance of supernatant was measured using UV-Vis spectroscopy (T80+UV/Vis spectrometer, PG Instrument Ltd., Leicestershire, UK) at 542 nm. All experiments were carried out in three repetitions. DI water and 0.9% NaCl solution were used as positive and negative control groups, respectively, and the hemolysis ratio percentage was calculated using Equation (1).

Hemolysis ratio% = ((A_sample_−A_negative control_)/(A_positive control_−A_negative control_)) × 100 (1)

where A_sample_, A_negative control_, and A_positive control_ are the absorbance values of the sample, negative control sample, and positive control samples, respectively.

#### 2.5.2. Blood Clotting Assay

The weighted 5 mg of each CNT, PDA-1st@CNT, PDA-3rd@CNT, and PDA-5th@CNT were placed in flat-bottom tubes for a blood-clotting study. After that, 64.8 μL of 0.2 M CaCl_2_ aqueous solution was added to 810 μL of fresh blood, and 270 μL of this solution was promptly dropped on the samples. After 10 min, 10 mL of DI water was progressively added to the tubes, and the tubes were centrifuged at 100 g for 1 min. Then, the supernatant solution was gently separated from the precipitated portion and diluted in another tube with 40 mL of distilled (DI) water. After 1 h, the absorbance of the supernatant was measured using UV-Vis spectroscopy at 542 nm. All tests were carried out three times. As a reference group, just 270 μL of fresh blood was utilized in 50 mL of DI water, and the following Equation (2) assessed the blood clotting index.

Blood clotting index = (A_sample_/A_reference_) × 100 (2)

where A_reference_ is the absorbance value of the reference.

### 2.6. Enzyme Inhibition Assay

For the enzyme inhibition studies, an alpha-glucosidase enzyme was used as a model enzyme, and 4-NPG was used as a substrate. The assay was carried out according to the literature with some modifications [49]. In short, the certain amount of prepared alpha-glucosidase enzyme solution in pH 6.8 potassium phosphate buffer (80 µL) was placed into 5 mL of pH 6.8 potassium phosphate buffer in a vial. After that, 0.5 mL of 4-NPG solution at 10 mM concentration was also placed into the same vial, and after a 20 min reaction, the obtained absorbance value at 405 nm was recorded as A_control_. On the other hand, 1 mL of CNT, PDA-1st@CNT, PDA-3rd@CNT, and PDA-5th@CNT solutions at 2 mg/mL concentration as an inhibitor was put into a vial with 4 mL of pH 6.8 potassium phosphate buffer and 80 µL of enzyme solution, separately. Then, 0.5 mL of 4-NPG solution at 10 mM concentration was added to each vial, and 20 min later, the absorbance values at 405 nm were read via UV-Vis spectrometer. The inhibition rate was calculated with Equation (3):

Inhibition% = (1 − (A_test_/A_control_)) × 100 (3)

where “A_test_” is the observed absorbance values at 405 nm from the sample solution added assay, and “A_control_” is the observed absorbance values at 405 nm from the enzyme assay.

Additionally, the various PDA@CNT solutions at concentrations of 0.25, 0.5, 1, 2, 4, and 6 mg/mL were also investigated to observe the effect of sample concentration on the inhibition of activity of alpha-glucosidase enzymes.

### 2.7. Antibacterial Assay

The antibacterial activity of CNT, PDA-5th@CNT, and protonated PDA-5th@CNT as PDA-5th@CNT-HCl were assessed using a macro-dilution test against *E. coli* ATCC 8739 as Gram-negative bacteria and *S. aureus* ATCC 6538 as Gram-positive bacteria, as described by Sun et al. [47]. The PDA-5th@CNT is protonated with the treatment of 100 mL of 1 M HCl solution at room temperature for 1 h. In the protonation process, 1 g of PDA-5th@CNTs was placed in 100 mL of 1 M HCl solution at room temperature for 1 h by mixing at 1000 rpm. Then, the protonated PDA-5th@CNTs were collected with centrifugation at 10,000 rpm and washed with water twice to remove excess HCl from the structure. The protonated and washed PDA-5th@CNTs were also dried with a freeze-dryer under similar conditions to those mentioned above.

The protonated PDA-5th@CNTs were washed with water twice to remove excess HCl from the structure before use in antibacterial tests. Bacteria were grown via overnight incubation at 35 °C in NB liquid medium, and their concentration was adjusted to about 0.5 × 10^8^ CFU/mL using the McFarland 0.5 standard. CNT, PDA-5th@CNT, and PDA-5th@CNT-HCl samples were weighted as 5, 10, 25, 50, and 100 mg, respectively, as an antimicrobial substance, and then sterilized by irradiation under a photoreactor at 420 nm for 2 min before analysis.

The macro-dilution assay was used to evaluate the minimum inhibitory concentration (MIC) of CNT, PDA-5th@CNT, and PDA-5th@CNT-HCl. In a liquid medium of NB, 9.9 mL of CNT, PDA-5th@CNT, and PDA-5th@CNT-HCl solutions were prepared at concentrations of 0.5, 1, 2.5, 5, and 10 mg/mL. Each tube received 100 µL of bacteria suspension at a concentration of 0.5 × 10^8^ CFU/mL, and the tubes were incubated at 35 °C for 24 h. The lowest concentration of CNT, PDA-5th@CNT, and PDA-5th@CNT-HCl containing tubes that prohibit observable bacterial growth was designated as the minimum inhibitory concentration (MIC). As a positive control, the antibiotic gentamicin was employed. All the tests were carried out three times.

## 3. Results

### 3.1. Multiple PDA Coating on CNTs

**Figure 1:**
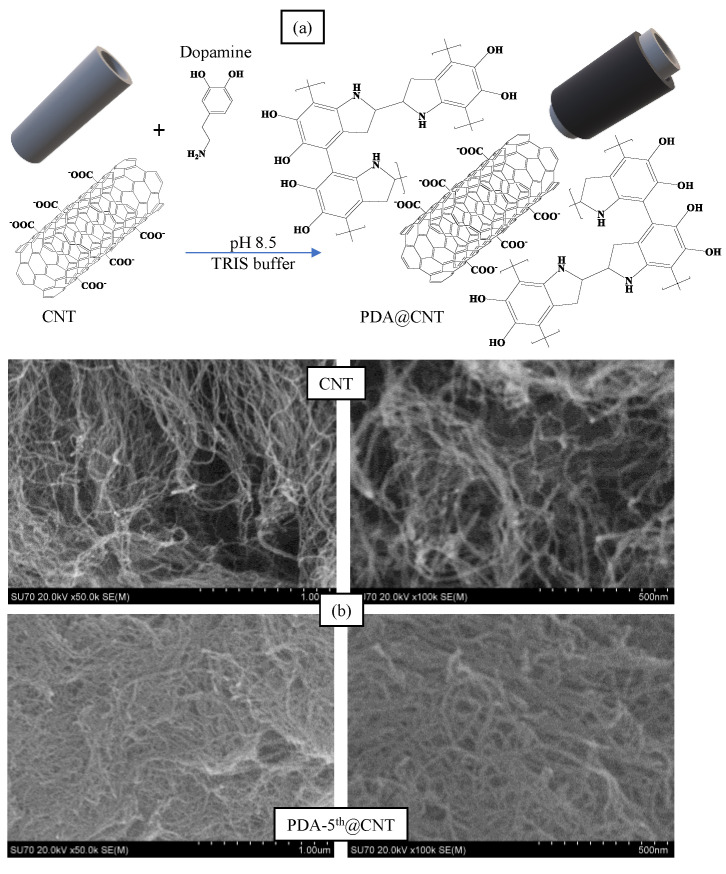
(**a**) The schematic presentation of coating process of Carbon nanotubes (CNTs) with polydopamine (PDA), and (**b**) Scanning Electron Microscopy (SEM) images of CNT and PDA-5th@CNT.

Alkaline medium has been utilized extensively in the literature for the self-polymerization of dopamine [50,51]. In a previous study by our group, it was observed that PDA, prepared in pH 8.5 TRIS buffer, started to collapse after 8 h of reaction time and was considered as a control experiment [46]. Therefore, in this study, the reaction time for the coating of CNTs with PDA was presumed as 6 h. The coating process of CNTs with PDA was carried out in a pH 8.5 TRIS buffer at room temperature. The schematic presentation of PDA coating on CNTs was illustrated in Figure 1a.

In brief, CNTs were placed into freshly prepared DA solution in pH 8.5 10 mM TRIS buffer at 2 mg/mL concentration. The mixture was stirred at 1000 rpm for 6 h, and the obtained PDA@CNTs were collected via centrifugation at 10,000 rpm. This cycle was repeated five times. The prepared PDA-coated CNTs were denoted as PDA-1st@CNT, PDA-2nd@CNT, PDA-3rd@CNT, PDA-4th@CNT, and PDA-5th@CNT, respectively. The SEM images of CNT and PDA-5th@CNT were also given in Figure 1b. It was clearly seen that the tubular structure of CNTs was maintained after even a 5th coating with PDA becoming slightly larger and hazy. It can be also seen from the SEM images that the PDA-coated CNTs can aggregate more than bare CNTs. It was reported that there was an increase in the hydrophobicity of CNT after PDA coating, and the increased hydrophobicity and aggregation enabled the utilization of PDA-coated materials’ new properties e.g., preventing corrosion and so on [43,52,53].

**Figure 2:**
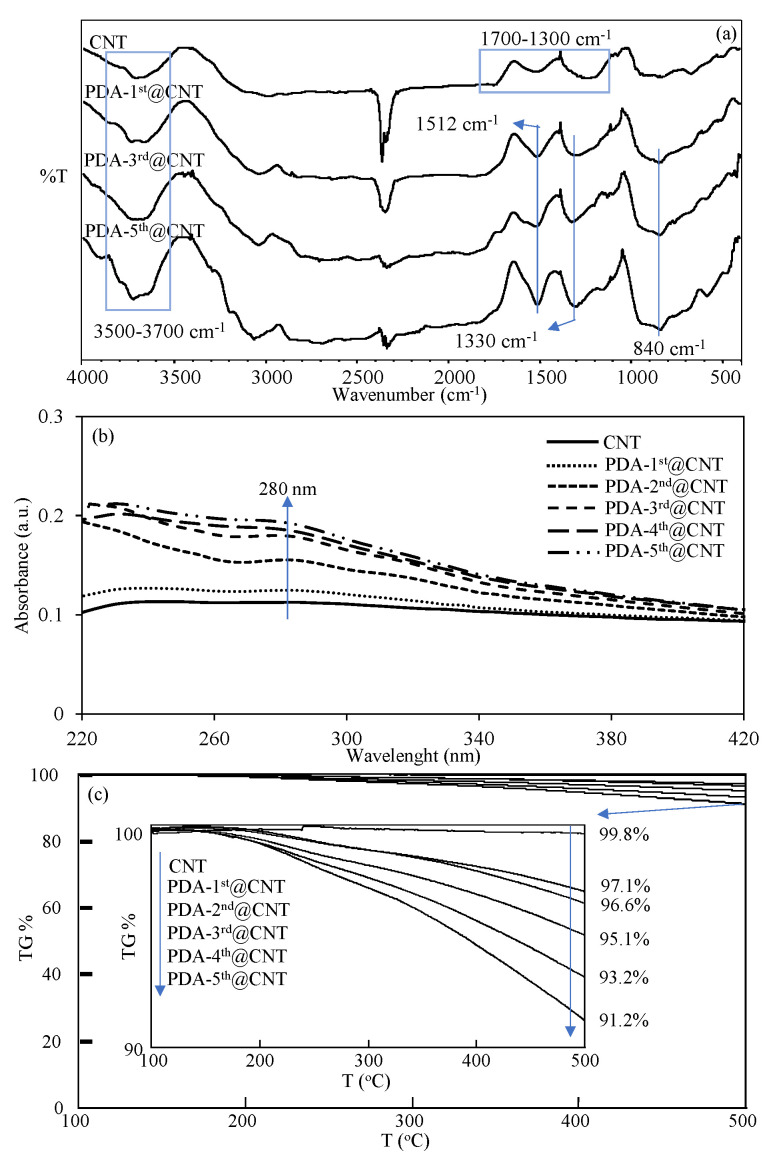
(**a**)FT-IR spectra, (**b**) UV-Vis spectra, and (**c**) TGA thermograms of CNT and PDA@CNTs with different numbers of PDA coatings.

The FT-IR spectra of CNT and PDA-coated PDA-1st@CNT, PDA-3rd@CNT, and PDA-5th@CNT were also compared in Figure 2a to confirm the success of the coating process of CNTs. It was clearly seen from the FT-IR spectra of the CNTs that the peaks in the 3700–3500 cm^−1^ range are due to the O-H vibrations from amorphous carbon, and the peaks between 1700 and 1300 cm^−1^ belong to the C=C skeleton stretching, C-C vibrations, and C-O stretching vibrations, respectively [54,55]. However, there are some distinct changes observed in the FT-IR spectrum of CNT after PDA coatings, which are the N-H bending at 1512 cm^−1^, O-H bending at 1330 cm^−1^, and C=C peaks at 840 cm^−1^, respectively [10]. Moreover, the UV-Vis spectra of bare CNT and PDA-coated CNTs were also recorded in 220–420 nm wavelength ranges to observe the characteristic absorbance of PDA, around 280 nm [43,56], and they are compared in Figure 2b after every coating.

It was clearly seen from the UV-Vis spectrum of bare CNT that there is no observed absorbance between 220 and 420 nm. On the other hand, an increase was observed with the increasing number of PDA coatings on CNTs, and it was observed that the visible difference around the 280 nm wavelength that came from PDA began to appear after the second coating. The slight increase in absorbance value around 280 nm with the increasing number of PDA coatings also confirmed the successful multiple coating process of CNTs with PDA. Moreover, to further corroborate the increased amount of PDA on CNTs with the multiple coating process, the TGA thermograms of bare CNT and PDA-coated CNTs were also compared, and the results are shown in Figure 2c. It is well-known that bare CNTs do not exhibit weight loss with the increase of temperature up to 500 °C [10]. Therefore, the increased amount of PDA on CNTs with the multiple coating process and even the amount of coated PDA on CNTs can be readily calculated by TGA analysis. In Figure 2c, the TGA thermograms of bare CNT, PDA-1st@CNT, PDA-2nd@CNT, PDA-3rd@CNT, PDA-4th@CNT, and PDA-5th@CNT were compared, and the TGA thermograms of CNT and PDA@CNTs exhibited increased weight loss percentages at 500 °C with the increased number of coatings. The inset in Figure 2c reveals the details of weight loss percentage values of bare CNT, PDA-1st@CNT, PDA-2nd@CNT, PDA-3rd@CNT, PDA-4th@CNT, and PDA-5th@CNT at 500 °C. It is clearly seen from inset of Figure 2c that there is almost no weight loss shown by bare CNT with 0.2% weight loss at 500 °C. On the other hand, the exhibited weight loss percentage values by PDA-1st@CNT, PDA-2nd@CNT, PDA-3rd@CNT, PDA-4th@CNT, and PDA-5th@CNT at 500 °C were determined as 2.9%, 3.4%, 4.9%, 6.8%, and 8.8%, respectively, affirming the increased extent of weight loss percentage values by the increased number of PDA coatings. Therefore, the percentage ratios of PDA on PDA@CNTs were calculated as 2.7, 3.2, 4.7, 6.6, and 8.6 for PDA-1st@CNT, PDA-2nd@CNT, PDA-3rd@CNT, PDA-4th@CNT, and PDA-5th@CNT, respectively. Gravimetrically, the total amounts of PDA on PDA@CNTs were determined as 27, 32, 47, 66, and 86 mg PDA on 1 g of PDA-1st@CNT, PDA-2nd@CNT, PDA-3rd@CNT, PDA-4th@CNT, and PDA-5th@CNT, respectively. From the TGA result, the weight losses were determined for PDA-coated CNT only for up to 500 °C heating. Therefore, these results are by no means the exact amounts of PDAs that are coated on CNTs, as one should keep in mind that by heating the PDA-coated CNTs up to higher values, the higher weight loss values for the CNTs with more PDA coatings should be expected.

**Figure 3:**
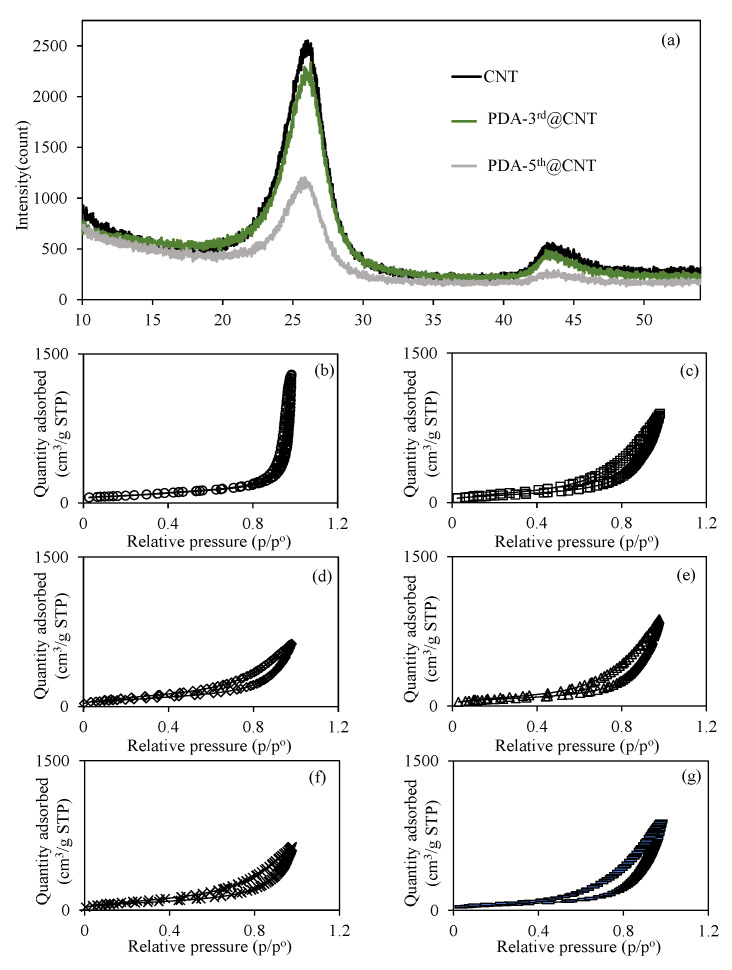
(**a**) XRD pattern of CNT and PDA@CNTs and the N_2_ adsorption/desorption curves of (**b**) CNT, (**c**) PDA-1st@CNT, (**d**) PDA-2nd@CNT, (**e**) PDA-3rd@CNT, (**f**) PDA-4th@CNT, and (**g**) PDA-5th@CNT.

Additionally, the XRD patterns of bare CNT, PDA-3rd@CNT, and PDA-5th@CNT were also compared in Figure 3a. The huge peak (002) at 25.9° and small peak (101) at 43.3° are indexed to the reflections of hexagonal graphite [57]. It was also clearly seen that the peaks assigned to reflections of hexagonal graphite appeared for all bare CNT, PDA-3rd@CNT, and PDA-5th@CNT. However, the intensity of peaks decreased with the increasing number of PDA coatings. The multiple PDA coatings of CNTs was also confirmed with the XRD analysis as well. In the literature, a coating of dopamine was applied once to the CNT for 24 h, and peaks were observed at 25° and 43° for CNTs and PDA coating on CNTs [58]. No change was observed in the intensity of the peaks with one layer of dopamine coating on CNTs, and it was mentioned that the crystallinity of graphite was not destroyed [58]. In this study, a slight difference in peaks was observed between CNT and PDA-3rd@CNT, while the difference was more clearly observed in PDA-5th@CNT, as can be seen in Figure 3a. The reduction in the XRD pattern of crystalline of graphite can be assumed to be due to the screening effect of multiple layers of PDA on the CNT.

On the other hand, the effect of multiple PDA coatings of CNTs on the surface area and porosity properties of CNTs was also investigated, and relevant N_2_ adsorption/desorption isotherms are given in Figure 3b–g for bare CNT, PDA-1st@CNT, PDA-2nd@CNT, PDA-3rd@CNT, PDA-4th@CNT, and PDA-5th@CNT, respectively. Figure 3b–g shows that the hysteresis loop in the N_2_ adsorption/desorption isotherms of the CNT and PDA@CNTs is in the range of 0.4 p/p° 0.9, indicating the presence of capillary condensation of N_2_ within mesopores. Furthermore, according to the International Union of Pure and Applied Chemistry (IUPAC), the adsorption type of CNT and PDA@CNTs should be type IV [58]. The calculated surface area, pore volume, and pore sizes values for CNT, PDA-1st@CNT, PDA-2nd@CNT, PDA-3rd@CNT, PDA-4th@CNT, and PDA-5th@CNT are also summarized and compared in Table 1.

It is apparent that the surface area of bare CNT of 263.9 m^2^/g was decreased with the multiple PDA coating process, and 197.0 m^2^/g surface area was calculated for PDA-5th@CNT. The decrease in the pore volume and pore size value of CNTs with the multiple PDA coatings indicate that the pores of CNTs are filling with PDAs during the coating process. The decrease in surface area of CNTs with the increase in the number of PDA coatings can also indicate the filling of CNT pores via the PDA coating procedure.

### 3.2. Effect of Multiple PDA Coating on Antioxidant Activity

**Figure 4:**
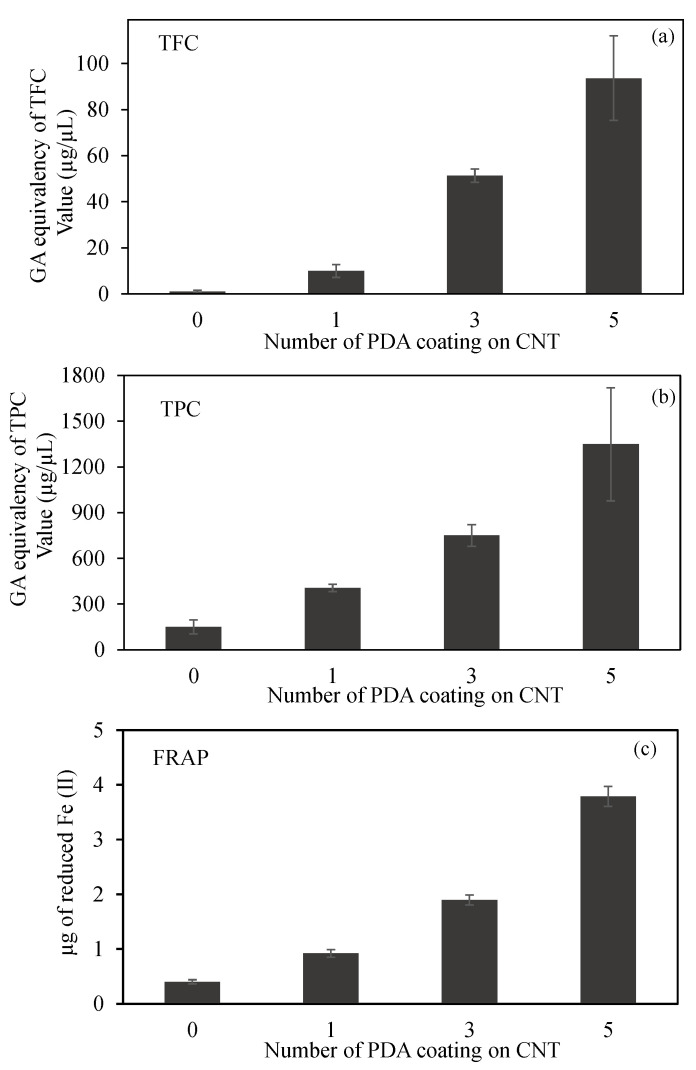
(**a**) Total flavonoid content (TFC) and (**b**) total phenol content (TPC) values in terms of gallic acid (GA) equivalency, and (**c**) ferric-reducing antioxidant power (FRAP) of CNT and PDA@CNTs at 2 mg/mL concentration.

Free radical formation and degradation, as well as numerous bioactivities such as material synthesis and degradation, energy transfer, and signal transduction occur continually throughout life [59]. Although free radicals serve vital biochemical roles, there is evidence that excessive quantities of them might disrupt biosystems and have unforeseeable effects [60]. For example, the increased amounts of reactive oxygen species (ROS) can interact with proteins, nucleic acids, and lipids to produce permanent tissue and organ damage, which can lead to cell aging, inflammation, and even cancer [61,62,63]. These circumstances are always inspiring new ideas for creating effective free radical scavengers to restore the overall radical equilibrium in biological systems. It has been reported in the literature and even in studies conducted by our group that PDA has antioxidant properties [46]. For this reason, the antioxidant properties of CNT and prepared PDA@CNTs were examined with three different tests, and the graphics of the relevant results are given in Figure 4.

The comparison of TFC test results of CNT, PDA-1st@CNT, PDA-3rd@CNT, and PDA-5th@CNT are given in Figure 4a. It was clearly seen that the bare CNTs did not exhibit any flavonoid content at 2 mg/mL concentration as expected. On the other hand, the GA equivalent of TFC values was increased with the increasing number of PDA coating on CNTs. TFC values of PDA-1st@CNT, PDA-3rd@CNT, and PDA-5th@CNT solution at 2 mg/mL concentration were found as 10.0 ± 2.8, 51.3 ± 2.8, and 93.7 ± 18.3 µg/mL GA equivalent flavonoid, respectively. The GA equivalent flavonoid content of PDA-3rd@CNT is almost five-fold higher than that of PDA-1^st^@CNT, and almost two-fold higher GA equivalent flavonoid content was determined for PDA-5th@CNT than for PDA-3rd@CNT.

A comparison of CNT, PDA-1st@CNT, PDA-3rd@CNT, and PDA-5th@CNT TPC test results is given in Figure 4b. According to Figure 4b, the bare CNTs exhibit 150.5 ± 45.9 µg/mL GA equivalent of TPC value at 2 mg/mL concentration, whereas the GA equivalent of the TPC value of bare CNT was three-fold increased after the 1st coating with PDA, and the GA equivalent of the TPC value for PDA-1st@CNT was determined as 450.5 ± 25.0 µg/mL at 2 mg/mL concentration. With an increasing number of coating processes, the GA equivalent of the TPC value almost linearly increased. It was observed that the PDA-3rd@CNT and PDA-5th@CNT exhibit 750.0 ± 69.9 and 1348.3 ± 371.7 µg/mL GA equivalent of TPC value at 2 mg/mL concentration, respectively. In the literature, it was stated that DP is an antioxidant molecule according to the TPC test [46]; this was corroborated with the literature, as the coating process did not destroy the antioxidant property of the PDA coating.

A FRAP test was also carried out to calculate the antioxidant capacity of CNT, PDA-1st@CNT, PDA-3rd@CNT, and PDA-5th@CNT, which are compared in Figure 4c. The FRAP test relies on antioxidant material to reduce the Fe(III)–TPTZ complex to a Fe(II)–TPTZ complex. As given in Figure 4c, in the presence of 2 mg/mL concentration of CNT solution, the reduced Fe(II) amount was calculated as 0.4 ± 0.04 µg. On the other hand, with the PDA coating on CNTs, the calculated reduced Fe(II) amount was increased to 0.9 ± 0.07 µg by PDA-1st@CNT at 2 mg/mL concentration. Moreover, it was clearly seen that with an increasing number of PDA coatings on CNTs, the reductive ability of the materials was increased, and the reduced Fe(II) amount was calculated as 1.9 ± 0.09 and 3.8 ± 0.18 µg for PDA-3rd@CNT and PDA-5th@CNT, respectively. Therefore, it can be assumed that the increasing antioxidant ability of PDA@CNTs with the increasing number of PDA coatings provides a controllable antioxidant activity for PDA@CNTs.

### 3.3. Blood Compatibility Changing via Multiple PDA Coatings

**Figure 5:**
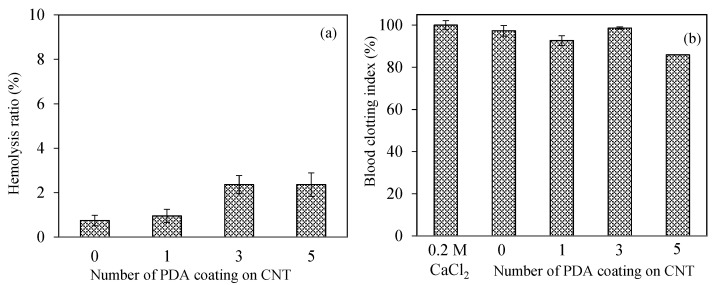
The assessment of hemocompatibility of bare CNTs and PDA@CNTs via (**a**) hemolysis and (**b**) blood-clotting assays.

The safety of nanomaterials should be examined in the design of biomedical materials for intravascular or wound-healing applications. Introducing nanomaterials that may pose coagulation and inflammatory components into the bloodstream and can be recognizable as an alien item that can be covered by serum proteins induces physiological responses such as inflammation, hemolysis, or blood-coagulation stimulation, according to the nature of materials [64]. The blood compatibility of CNT, PDA-1st@CNT, PDA-3rd@CNT, and PDA-5th@CNT was determined by hemolysis and blood compatibility tests, and the results are illustrated in Figure 5. A hemolytic ratio of up to 5% is considered to be hemocompatible for related applications [65]. As can be seen in Figure 5a, all forms of CNT, PDA-1st@CNT, PDA-3rd@CNT, and PDA-5th@CNT showed non-hemolytic behavior with a maximum 2.4 ± 0.5% hemolysis ratio up to 0.5 mg/mL concentration. In addition, the 0.7 ± 0.2% hemolysis ratio of bare CNTs at 0.5 mg/mL concentration was slightly increased with the PDA coating, and the hemolysis ratio was calculated as 0.9 ± 0.3 for PDA-1st@CNT and became almost stable for PDA-3rd@CNT and PDA-5th@CNT with 2.4 ± 0.4, and 2.4 ± 0.5% hemolysis ratios, respectively. These results represent that the hemocompatibility of CNT, PDA-1st@CNT, PDA-3rd@CNT, and PDA-5th@CNT was slightly decreased depending on the number of PDA coatings on CNTs and suggest that these materials are totally safe for intravascular application and/or wound-healing applications up to 0.5 mg/mL concentration.

The other blood compatibility tests such as the blood clotting indexes of CNT, PDA-1st@CNT, PDA-3rd@CNT, and PDA-5th@CNT are given in Figure 5b. Blood coagulation is a complicated series of interconnections between the endothelium and coagulation factors, often known as “coagulation stunts”, which involve intrinsic and extrinsic pathways [65]. Blood coagulation is targeted at being regulated locally started. Cascade coagulation activation can lead to intravascular thrombination development in uncontrolled or unwanted conditions, leading to adverse/fatal outcomes [66]. In the processes of coagulation, even modest changes in the natural function of blood components, e.g., foreign particles can promote coagulation by the complex effects of responses and amplifications [65,66]. The control group, 270 µL of 0.2 M CaCl_2_ solution with fresh blood added, exhibited no coagulation effect after 10 min of interaction with fresh blood. It was also observed that blood was coagulated after 20 min of interaction with 270 µL of 0.2 M CaCl_2_ solution. Moreover, it was clearly seen that there are more than 90% blood clotting indexes observed for CNT, PDA-1st@CNT, and PDA-3rd@CNT at 0.5 mg/mL concentration, respectively. On the other hand, the 5th coating of CNT with PDA slightly decreased the clotting index, and the blood clotting index was calculated as 85.5 ± 0.5% for PDA-5th@CNT at 0.5 mg/mL concentration. These results indicate that the CNT structure’s PDA coating activates the clotting capacities of the PDA@CNTs. The safety of these materials in interacting blood applications is shown by the lower hemolysis ratio and by the high index of blood clotting values for CNT, PDA-1st@CNT, PDA-3rd@CNT, and PDA-5th@CNT at 0.5 mg/mL concentrations.

### 3.4. PDA-Coated CNTs as Antidiabetic Drug

**Figure 6:**
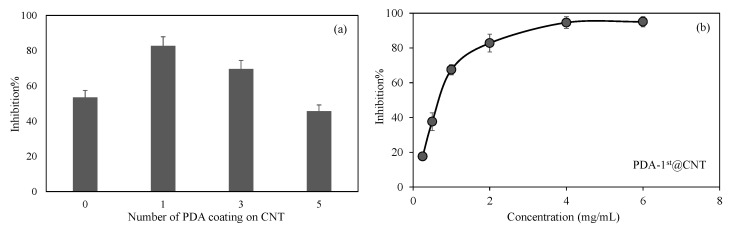
(**a**) Inhibitory effect of CNT and PDA@CNTs on the activity of alpha-glucosidase enzymes at 2 mg/mL concentration and (**b**) concentration-dependent inhibition of activity of alpha-glucosidase enzyme in the presence of PDA-1st@CNTs between 0.25 and 6 mg/mL concentration.

Many studies have found that the global prevalence of diabetes has grown in recent years, with estimates that the number of diabetic people would reach 642 million by 2040 [67]. If diabetes is not treated early on, it can lead to a variety of problems as well as a high rate of death and morbidity over time [68]. Although there are numerous types of medicines for the treatment of Type 2 diabetes, it has been noted that they have some safety restrictions as well as certain drawbacks of marketed pharmaceuticals such as poor patient compliance and cost [69]. This highlights the need for new medication classes with improved efficacy, safety, patient compliance, and cost-effectiveness. However, numerous studies in recent years have shown that alpha-glucosidase enzyme inhibitors are critical in the proper management of Type 2 diabetes [70,71,72]. Alpha-glucosidase is a pancreatic enzyme involved in the conversion of 80–90% of carbs into glucose. The alpha-glucosidase enzyme releases glucose into the bloodstream, causing hyperglycemia, which worsens diabetes patients’ symptoms and speeds up complications. These enzymes are inhibited, which helps avoid postprandial hyperglycemia and the production of glycated end products [67,68]. The alpha-glucosidase inhibitory effect of PDA particles was successfully reported via a previously study of our group [46]. In the literature, there are some studies that also reported that CNT inhibits some enzymes such as Cytochrome P450 enzyme [73], pepsin [74], and alpha-chymotrypsin [75]. Therefore, the inhibitory effects of CNT, PDA-1st@CNT, PDA-3rd@CNT, and PDA-5th@CNT on the activity of alpha-glucosidase enzymes were also in vitro studied and compared with each other, and the results are demonstrated in Figure 6.

In Figure 6a, the inhibitory effect of CNT, PDA-1st@CNT, PDA-3rd@CNT, and PDA-5th@CNT solution at 2 mg/mL concentration on the activity of alpha-glucosidase enzyme was given. It was observed that the alpha-glucosidase enzyme activity was inhibited via bare CNT at 2 mg/mL concentration with 53.5 ± 3.9% inhibition. On the other hand, for the same concentrations of PDA-1st@CNT, PDA-3rd@CNT, and PDA-5th@CNT solution, the enzyme inhibitions were determined as 82.8 ± 5.1%, 69.6 ± 4.8%, and 45.6 ± 3.6%, respectively. The higher enzyme inhibition was observed for PDA-1st@CNT. It is understood from these results that there is a cumulative effect of CNT and dopamine together in the first coating. However, as the number of coatings increases, the effect of enzyme inhibition decreases. This situation may be due to two reasons. Firstly, it is possible that CNTs’ inhibition properties are masked with the presence of the first layer of PDA, and as the number of PDA layers is increased, the PDA’s ability to inhibit the enzyme is decreased. Secondly, with the higher number of PDA coatings on CNT, the aggregation through the increased number of coatings is possible, which leads to less interactions with the enzyme, hence leading to the decrease in overall inhibition percentage with respect to the 1st and 3rd coatings.

Therefore, the effect of concentration of PDA-1st@CNTs was also investigated, and a related graph is given in Figure 6b. It was observed that the increase in the concentration of PDA-1st@CNTs in the 0.25–6 mg/mL range was found to increase the inhibition percentage values of activity of alpha-glucosidase enzyme. The alpha-glucosidase enzyme activity was almost inhibited 80% in the presence of PDA-1st@CNT at 0.25 mg/mL concentration, and the observed inhibition percentage was increased more than 90% at 4 mg/mL and 6 mg/mL concentration of PDA-1st@CNTs. It can clearly be deduced from Figure 6b that the tunable inhibition of alpha-glucosidase enzyme is possible with a concentration-dependent use of PDA-1st@CNTs as an inhibitor. These results show that the prepared PDA@CNTs possess great potential as an antidiabetic drug; however, in vivo studies should be carried out to ascertain certain outcomes.

### 3.5. PDA@CNTs as Antibacterial Materials

With the obtained results of the antioxidant, blood-compatible, and enzyme-inhibitory properties of PDA@CNTs, the possible antibacterial effect of PDA@CNT was also investigated. The antibacterial studies were carried out via the higher PDA containing CNTs such as PDA-5th@CNT and its protonated form PDA-5th@CNT-HCl. The antibacterial effect of CNT, PDA-5th@CNT, and PDA-5th@CNT-HCl was compared against Gram-negative *E. coli* and Gram-positive *S. aureus* bacteria via macro-dilution tests. The results are summarized in Table 2.

It was clearly seen from Table 2 that the CNT and PDA-5th@CNT are not antibacterial materials up to 10 mg/mL concentration. On the other hand, the PDA-5th@CNT-HCl at 10 mg/mL concentration prevents the growth of Gram-negative *E. coli* and Gram-positive *S. aureus* bacteria. Negatively charged polyphosphates in the membrane structure of Gram-negative and Gram-positive species of bacteria offer a high interaction capacity with PDA-5th@CNT-HCl due to their increased cationic nature, which can be useful in the antibacterial application of PDA-5th@CNT-HCl materials.

## 4. Conclusions

The multiple coating of CNTs with PDA was successfully carried out by the simple self-polymerization of DA molecules in a 10 mM TRIS buffer at pH 8.5 in the presence of CNTs. The amount of coated PDA on CNTs was calculated via TGA measurements. The amounts of PDA on CNTs for each coating after five consecutive coatings of CNTs via DA self-polymerization were determined as 27, 32, 47, 66, and 86 mg PDA for 1 g CNT as PDA-1st@CNT, PDA-2nd@CNT, PDA-3rd@CNT, PDA-4th@CNT, and PDA-5th@CNT, respectively. The increased amount of PDA on CNTs after a multiple coating process revealed the decreased surface area of bare CNTs from 263.9 m^2^/g to 256.7, 251.5, 249.2, 247.7, and 197.0 m^2^/g after the 1st, 2nd, 3rd, 4th, and 5th coatings of PDA. The decrease in pore size was also observed; e.g., the pore size of CNTs from 26.2 to 10.2 nm after five consecutive coating processes with PDA was measured, implying the fillings of the pores of CNTs with PDA. The antioxidant ability of prepared PDA@CNT structures was confirmed via three different antioxidant assays. Each assay showed that by increasing the amount of PDA coating on CNTs via multiple coating processes, the antioxidant ability of PDA@CNTs was increased. The simple multiple coating of CNTs with PDA via the self-polymerization of DA in this study recommends the use of these materials as controllable antioxidant ability. The hemocompatibility of PDA@CNTs assessed by in vitro hemolysis and blood-clotting assays also showed a safe use of PDA@CNTs for applications involving direct blood contact applications e.g., hemodialysis, medication administration, etc. at concentrations ≥0.5 mg/mL. However, a more comprehensive analysis should be carried out before the deployment of PDA@CNTs for in vivo research to ascertain the intended use to determine appropriate dose levels. In addition, the concentration-dependent inhibitory properties of the prepared PDA@CNTs on the activity of the alpha glucosidase enzyme showed their possible use as a drug in the treatment of Type 2 diabetes. Moreover, the prepared PDA-5th@CNT can be also used in preventing bacterial growth at 10 mg/mL concentration after protonation with HCl. It was reported that the functionalized CNTs could be used in cancer therapy and diagnosis [76]. Additionally, PDA was also reported as a resourceful material for different biomedical applications [77]. Here, by combining PDA and CNT, it was demonstrated that the different layer PDA-coated CNTs can be used as drug delivery systems, antimicrobial and antioxidant agents, and as biomedicine itself due to their possible influence on macrophages [44]. Moreover, because of the antioxidant properties of the PDA-coated CNTs, these materials can be readily employed in cosmetic industries. As the biomedical and industrial processes based on the multifunctional design of micro/nano interfaces have improved, safe and green materials are of major relevance from a natural and sustainable source; thus, PDA@CNTs made from natural, biocompatible, and renewable precursors are promising materials to offer a wide variety of biological, diagnostic, and therapeutic applications as porous and tunable interfaces.

## Figures and Tables

**Table 1:** The effect of multiple coatings of PDA on the CNTs’ surface area, pore volume, and pore size values.

Materials	Surface Area (m^2^/g)	Pore Volume (cm^3^/g)	Pore Size (nm)
CNT	263.9	2.0	26.2
PDA-1st@CNT	256.7	1.3	13.2
PDA-2nd@CNT	251.5	1.3	12.4
PDA-3rd@CNT	249.2	1.3	12.1
PDA-4th@CNT	247.7	0.9	11.1
PDA-5th@CNT	197.0	0.9	10.2

**Table 2:** Minimum inhibition concentration (MIC) values of CNT-based materials against *E. coli* ATCC 8739 (Gram−) and *S. aureus* ATCC 6538 (Gram+).

Materials	* MIC (mg/mL)	
	*E. coli*	*S. aureus*
CNT	N.D.	N.D.
PDA-5th@CNT	N.D.	N.D.
PDA-5th@CNT-HCl	10	10

* MIC values of gentamicin for *E. coli* and *S. aureus* are 0.001 and 0.002 mg/mL, respectively.
